# A Ground-Based Near Infrared Camera Array System for UAV Auto-Landing in GPS-Denied Environment

**DOI:** 10.3390/s16091393

**Published:** 2016-08-30

**Authors:** Tao Yang, Guangpo Li, Jing Li, Yanning Zhang, Xiaoqiang Zhang, Zhuoyue Zhang, Zhi Li

**Affiliations:** 1 ShaanXi Provincial Key Laboratory of Speech and Image Information Processing, School of Computer Science, Northwestern Polytechnical University, Xi’an 710129, China; liguangponwpu@gmail.com (G.L.); ynzhangnwpu@gmail.com (Y.Z.); vantasy@mail.nwpu.edu.cn (X.Z.); zhangzzy@mail.nwpu.edu.cn (Z.Z.); zLeewack@163.com (Z.L.); 2 School of Telecommunications Engineering, Xidian University, Xi’an 710071, China; jinglixd@mail.xidian.edu.cn

**Keywords:** UAV, automatic landing, infrared camera array, vision-based navigation

## Abstract

This paper proposes a novel infrared camera array guidance system with capability to track and provide real time position and speed of a fixed-wing Unmanned air vehicle (UAV) during a landing process. The system mainly include three novel parts: (1) Infrared camera array and near infrared laser lamp based cooperative long range optical imaging module; (2) Large scale outdoor camera array calibration module; and (3) Laser marker detection and 3D tracking module. Extensive automatic landing experiments with fixed-wing flight demonstrate that our infrared camera array system has the unique ability to guide the UAV landing safely and accurately in real time. Moreover, the measurement and control distance of our system is more than 1000 m. The experimental results also demonstrate that our system can be used for UAV automatic accurate landing in Global Position System (GPS)-denied environments.

## 1. Introduction

Unmanned air vehicles (UAVs) have become more and more prevalent recently. However, one of the most difficult challenges for both manned and unmanned aircraft is safe landing. A significant number of accidents happens during the landing phase due to inexperience of pilots or sudden changes in the weather dynamics, thus automatic landing systems are required to land UAVs safely [1]. How to develop autonomous landing systems has been a hot issue of the current UAV researches, it is a challenge for its high requirements of reliability and accuracy.

Several methods of the control of the UAV have been developed, such as PID control [2], backstepping [3,4,5], $H_{\infty }$ control [6], sliding mode control [7], fuzzy control [8], and model based fault tolerant control [9]. The traditional navigation systems for landing which have been on the UAV mainly include Inertial Navigation System (INS), Global Position System (GPS), INS/GPS combined navigation system, Global Navigation Satellite System (GNSS) and so on [10]. One of the most commonly used methods is the GPS/INS integrated navigation, but GPS signals are easily blocked, lower height accuracy [11], and INS tends to drift because the errors accumulate over time [12].

As described previously, the height measurement from the GPS is usually inaccurate, which is easy to cause a crash, thus other sensors may be needed like radar altimeter. The most important is that the GPS signals may not be always available, the automatic landing may not be possible in many remote regions or GPS-denied environments. At this time, the advantages of vision-based automatic landing method is particularly important.

In recent years, new measurement systems which take visual sensors as cores have been applied more and more widely and expanded to the UAV automatic landing [10]. Guo et al. [12] proposed a vision-aided landing navigation system based on fixed waveband guidance illuminant using a single camera. Ming et al. [13] adopted a vision-aided INS method to implement the UAV auto landing navigation system. Vladimir et al. [14] presented a robust real-time line tracking algorithm for fixed-wing aircraft landing. Abu-Jbara [15] and Cao et al. [16] studied the airport runway in natural scenes, while Sereewattana et al. [17] provided a method to find the runway by adding four different color marks, Zhuang et al. [18] used the two edge lines on both sides of the main runway and the front edge line of the airport to estimate the attitude and position parameters. Li et al. [19] extracted three runway lines in the image using Hough transform method to estimate the pose of the UAV. Barber et al. [20] used a visual marker to estimate the roll and pitch for flight control. Huh et al. [11] proposed a vision-based automatic landing method which used a monotone hemispherical airbag as a marker. Lange et al. [21] adopted an adaptive thresholding technique to detect a landing pad for the multirotor UAV automatic landing. Miller et al. [22] proposed an navigation algorithm based on image registration. Daquan et al. [23] estimated aircraft’s pose using extended Kalman filter (EKF).

In the papers mentioned above, most of them are based on a downward-looking camera to recognize the airport runway, the artificial markers or the natural markers in the image, etc. However, the stabilizing controllers based on monocular camera are subject to drift over time [24], the field of view can be temporarily occluded and the illumination conditions might change drastically within a distance of a few meters [25], and extracting the symbols such as runway and the performance of the image-matching algorithm are greatly influenced by imaging circumstance, and it is hard to extract the symbols correctly and the performance of the image-matching algorithm may be unsatisfied in complicated situations such as rain, fog and night [10]. Compared with onboard navigators, the ground-based system possesses stronger computation resources and enlarges the search field of view. Wang et al. [26] used a ground USB-camera to track a square marker patched on the micro-aircraft. Martinez et al. [27] designed a trinocular system, which is composed of three FireWire cameras fixed on the ground, to estimate the vehicle’s position and orientation by tracking color landmarkers on the UAV. Researchers in Chiba University [28] designed a ground-based stereo vision system to estimate the three dimensional position of a quadrotor. The continuously adaptive mean shift algorithms was used to track the color based object. Abbeel et al. [29] achieved autonomous aerobatic flights of an instrumented helicopter using two Point Grey cameras with known positions and orientations on the ground. The accuracy of the estimates obtained was about 25 cm at about 40 m distance from the cameras.

There is one similar automatic landing system called OPATS (Object Position and Tracking System), which is developed by RUAG for the Swiss Air force in 1999 [30]. However, here are several different aspects compared our system with OPATS. First of all, the theories are different. OPATS is a laser-based automatic UAV landing system that continuously measures the dynamic positions of the object of the interest, using one laser sensor with tripod, etc. While the proposed landing system is based on stereo vision using infrared camera array. Thus their location theories are different. The location theories of the OPATS is based on the infrared laser beam reflected by the retroreflector on the UAV, while our infrared camera array system is based on the theory of binocular positioning. Secondly, the adopted equipment is different. The equipment on the ground of OPATS mainly includes: a standalone laser sensor, electronics unit, battery. The equipment on the ground of our landing system mainly include: infrared laser lamp, infrared camera array, camera lens and optical filter. The equipment fixed on the UAV of OPATS: passive optical retroreflector. The equipment fixed on the UAV of our landing system: near infrared laser lamp. Most importantly, OPATS can only guide one UAV landing at the same time, while the proposed landing system in this paper covers wide field of regard, which has the ability to guide several UAVs landing at the same time. And this problem could be described as “multi-object tracking”, which is very important as the development of UAV. A pan-tilt unit (PTU) is employed to actuate the vision system in [31]. Although it can be used under all weather conditions and around the clock, the limited baseline has resulted in short detection distance. To achieve long range detection and cover wide field of regard, a newly developed system has been designed in [32], which is the most similar works to ours. Two separate sets of PTU are mounted integrated with visible light camera on both sides of the runway instead of their previous stereo vision system, which is able to detect the UAV around 600 m. However, the detection results are unsatisfied when the background become cluttered.

**Figure 1:**
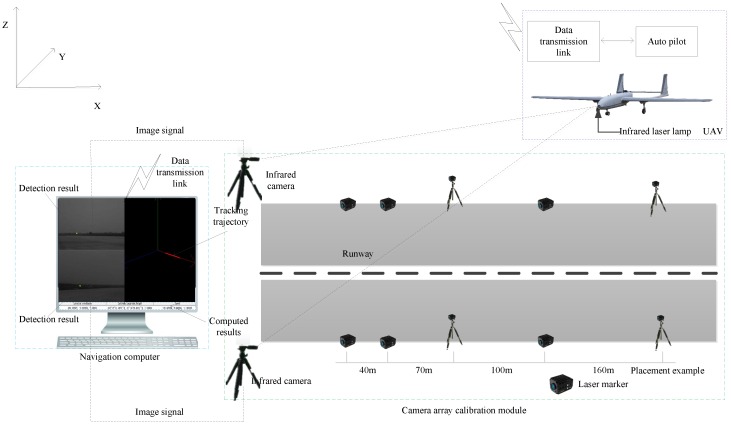
Infrared camera array system.

In order to land the UAVs safely in GPS-denied environment, a novel ground-based infrared camera array system is proposed in this paper as shown in Figure 1. The direct detection of the UAV will be limited by detection range, thus a near infrared laser lamp is fixed on the nose to instead the position of the UAV, which simplifies the problem and improve the robustness of the system. Two infrared cameras are located on the two sides of the runway respectively to capture flying aircraft images, which are processed by the navigation computer. After processing, the real time position and speed of the UAV are sent to the UAV control center, and the detection results, tracking trajectory and three-dimensional localization results are displayed in real time. The infrared camera array system cooperated with the laser lamp can effectively suppress the interference of the light and it can be employed around the clock under all weather condition.

In this paper, we present the design and construction of hardware and software components to accomplish autonomous accurate landing of the UAV. Our work mainly makes three contributions:

- We propose a novel infrared camera array and near infrared laser lamp based cooperative optical imaging method, which greatly improve the detection range of the UAV.
- We design a wide baseline camera array calibration method. The method presented could achieve high precision calibration and localization results.
- We develop a robust detection and tracking method for near infrared laser marker.

The proposed system is verified using a middle-sized fixed-wing UAV. The experiments demoustrate that the detection range has been greatly improved, which is more than 1000 m, and a high localization accuracy is achieved. The system has also been validated in the GPS-denied environment and the UAV is guided to land safely.

The rest of the paper is organized as follows. In Section 2, we describe the design and methodology of our landing system. Section 3 describes the experimental results and followed the conclude results in Section 4.

## 2. Landing System

**Figure 2:**
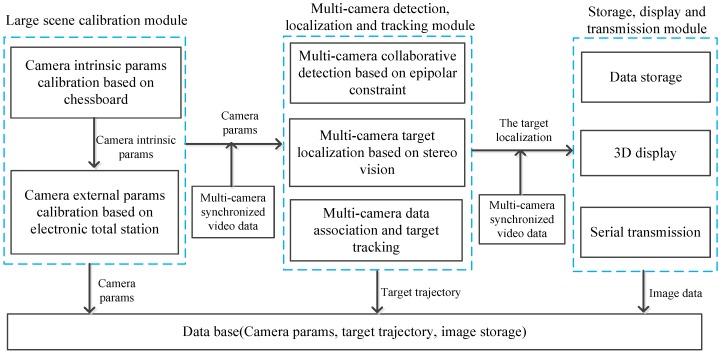
The framework of the infrared camera array system.

**Figure 3:**
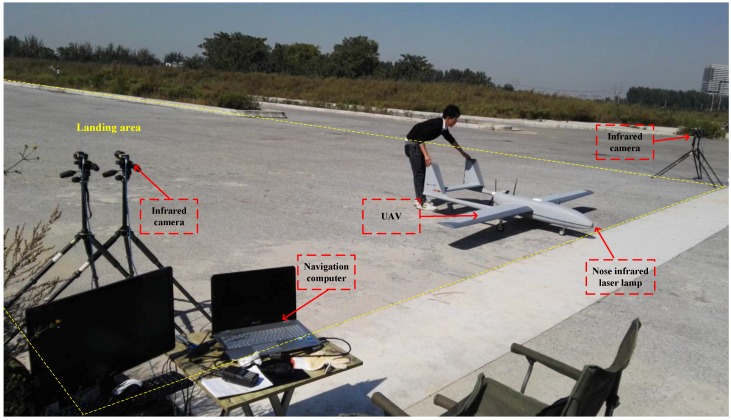
Ground-based landing system.

This paper focuses on the content of the vision-based landing navigation. Figure 2 shows the system framework and the complete experimental setup is shown in Figure 3.

We adopt a vision-based method, and how to get clear images of the target at long-distance is our first task. In our system, the light source is carried on the nose to instead of the position of the UAV. After many tests and analyses, we finally choose the near infrared laser lamp and infrared camera array to form the optical imaging module.

The accuracy of the camera array parameters directly determines the localization accuracy, while the traditional method of measuring binocular calibration accuracy will serious decline in long-distance. In this paper, a calibration method for infrared camera array is proposed, which can achieve a high calibration accuracy.

When the UAV is landing from a long distance, the laser marker fixed on the UAV presents the characteristics of small target. Besides, the target may be influenced by the strong sunlight, signal noise and other uncertain factors in the actual situation. In this paper, these problems are discussed in detail and solved effectively.

### 2.1. Optical Imaging Module

For vision-based UAV automatic landing systems, one of the basic steps is to construct the optical imaging module. A good optical imaging module will make the target in the image prominent, simplify the algorithm, guarantee the stability of the system, etc. The main components of our optical imaging module are near infrared laser lamp, infrared camera array and optical filter.

The results of the direct detection of the UAV are usually not robust, especially at long distance, which is greatly affected by background. Thus the detection range is usually limited. To improve the detection range, we carefully design the vision system by introducing the light source, which plays an important role in optical imaging module. It directly affects the quality of the image, and then affects the performance of the system. In our system, the light source is carried on the nose to replace the position of the UAV. The function of the light source is to obtain a clear image with a high contrast. One of the required characteristics of the selected light source is insensitive to visible light. Taking the factors of the wind into consideration, the light source should have the robustness of multi-angle. We have conducted a lot of comparative tests of different light sources, and finally choose the near infrared laser lamp. The near infrared laser lamp parameters are shown in Table 1. The illumination distance of the near infrared laser lamp is more than 1000 m, which guarantees the detection range. The wavelength of the near infrared laser lamp is 808 ± 5 nm. Its weight is only 470 g, which is suitable to be fixed on the UAV.

Our ground-based system is mainly designed for fixed-wing UAVs which have a high flight speed and landing height. The function of the camera is to capture images of the near infrared laser lamp fixed on the UAV. So the camera needs to have a high sampling rate for the dynamic target, and should have enough resolution so that the target can still be clearly acquired at a long distance. Considering these, we finally choose the infrared camera of Point Grey GS3-U3-41C6NIR-C with 2048×2048 pixels. In order to ensure the camera resolution and spatial location accuracy, we select the the camera lens of Myutron HF3514V with focal length of 35 mm. The cameras are fixed on each side of the runway as shown in Figure 3, which has a wide baseline. The camera parameters are shown in Table 2 and the camera lens parameters are shown in Table 3. The maximum frame rate of the camera is 2048×2048 at 90 fps, which meets the need of the proposed system. The maximum magnification of the camera lens is 0.3× and its TV distortion is −0.027%.

**Figure 4:**
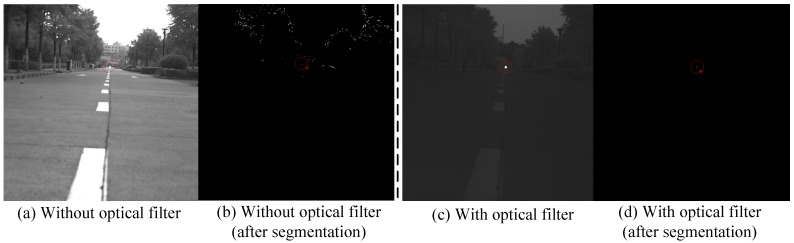
The ability to resist interference in complex environments (650 m).

In order to increase the anti-interference ability of the optical imaging module, a kind of Near-IR interference bandpass filter is adopted. The wavelength of the optical filter is 808 nm, of which the signal attenuation is small. The filter is fixed in front of the camera lens, so the camera is only sensitive to the wavelength of 808 nm. The emission wavelength of the near infrared laser lamp fixed on the UAV is 808 nm. The filter is a component of the infrared camera array. The cooperation of the infrared camera array and the light source could guarantee distinct imaging of the near infrared laser lamp and effectively avoid interferences of complicated background. Thus the robustness and the detection range of the system are both greatly improved. As shown in Figure 4, we can see that the filter could get rid of almost every interferential signal successfully. The optical filter parameters are shown in Table 4.

### 2.2. Large Scale Outdoor Camera Array Calibration

The process of calibration is to estimate the intrinsic parameters and extrinsic parameters of the cameras in the array system.

To get high localization accuracy, precise camera parameters are needed. Classical camera calibration methods include Weng’s [33], Zhang’s [34], etc. For those traditional camera calibration methods, the reference points or lines must be distributed in space or in the calibration image rationally, which is easy to be constructed indoors or if the view field is not large. To obtain the precise camera parameters in large scale outdoors, a new camera array calibration method is presented.

Chessboard pattern is adopted to obtain intrinsic parameters. As described before, two infrared cameras are located on both sides of the airport runway to enlarge the baseline, which contributes to promoting the localization accuracy. However, it brings difficulties to the calibration of the external parameters. Thus a novel camera external parameters calibration method based on electronic total station is presented here. The parameters of the electronic total station used in our system are shown in Table 5. The measuring distance of the electronic total station could reach 2000 m and its ranging accuracy is ±(2 mm + 2 ppm). And its angle measurement accuracy is $\pm 2^{\prime \prime }$. We can see that the electronic total station has ensured a high accuracy of measurement. To get precise external parameters, ten near infrared laser lamps are also putted on both sides of the runway as control points as shown in Figure 1, one placement example of the distance between each pair of the near infrared laser lamps is presented here. Six of them are located closer to the ground, and four of them are located about 2.0 m height from the ground. The external parameters calibration method mainly includes the following steps:

(1) Establish a world coordinate system.
(2) Measure the precise world coordinates of the control points using the electronic total station.
(3) Extract the projections of the control points from the two calibration images.
(4) Accurately estimate the spot center coordinates of the control points using bilinear interpolation.
(5) Obtain the initial external parameters by DLT (Direct Linear Transform) algorithm [35].
(6) Generate the final calibration results by LM (Levenberg-Marquardt) algorithm [35].

It is necessary for centroids to be extracted after the region is determined in step 4. In order to improve the accuracy and stability, bilinear interpolation method are used before calculating the coordinate of the spot center:

(1) $$\left\{\begin{array}{c} g(i+x,j)=g(i,j)+x[g(i+1,j)-g(i,j)]\\ g(i,j+y)=g(i,j)+y[g(i,j+1)-g(i,j)]\\ \begin{array}{cc} g(i+x,j+y)= & x[g(i+1),j)-g(i,j)]+\\ & y[g(i,j+1)-g(i,j)]+\\ & xy[g(i+1,j+1)+\\ & g(i,j)-g(i+1,j)-\\ & g(i,j+1)]+g(i,j) \end{array} \end{array}\right.,$$

where g(i,j) is the gray value of the point (i,j), x,y∈(0,1). Then the subpixel of the spot center $\left(x_{0},y_{0}\right)$ is calculated by:

(2) $$\left\{\begin{array}{c} x_{c}=\sum_{i\;=\;x_{b}}^{x_{e}}x_{i}\cdot w(x_{i},y_{i})/\sum_{i\;=\;x_{b}}^{x_{e}}w\left(x_{i},y_{i}\right)\\ y_{c}=\sum_{i\;=\;y_{b}}^{y_{e}}y_{i}\cdot w(x_{i},y_{i})/\sum_{i\;=\;y_{b}}^{y_{e}}w\left(x_{i},y_{i}\right) \end{array}\right.and$$

(3) $$\left\{\begin{array}{cc} w\left(x_{i},y_{i}\right)=g\left(x_{i},y_{i}\right) & g(x_{i},y_{i})\geq T0\\ w(x_{i},y_{i})=0 & g(x_{i},y_{i})<T0\\ x_{b}=x_{0}-T1\\ x_{e}=x_{0}+T1\\ y_{b}=y_{0}-T1\\ y_{e}=y_{0}+T1 \end{array}\right.,$$

where $x_{i}$ and $y_{i}$ are the values of horizontal and vertical coordinates of the point $\left(x_{i},y_{i}\right)$, T0 and T1 are the thresholds.

### 2.3. Target Detection, Localization and Tracking

***Target Detection:*** Because of the obviously different grayscale between the target and background, we directly acquire the foreground image of the candidate targets after a simple morphological pre-processing, and then the foreground cluster is carried to get the coordinates of the candidate targets in the image. If pixel distance $f_{pd}\left(p_{i},p_{j}\right)$ is less than foreground clustering window *J*, then clustered as a class $x_{i}\left(i\geq 0\right)$. We regard the image centroid coordinate of each cluster as the coordinate of the candidate target in the image. The pixel distance is defined as:

(4) $$f_{pd}\left(p_{i},p_{j}\right)=\sqrt{{\left(p_{i}^{x}-p_{j}^{x}\right)}^{2}+{\left(p_{i}^{y}-p_{j}^{y}\right)}^{2}},$$

where $p_{i}$ and $p_{j}$ are image pixels, $\left(p_{i}^{x},p_{j}^{x}\right)$ and $\left(p_{i}^{y},p_{j}^{y}\right)$ are pixel coordinates of $p_{i}$ and $p_{j}$ respectively.

To determine the corresponding relationship of the candidate targets and remove the false targets, epipolar geometry constraints between the two cameras are used. Epipolar geometry between the two cameras refers to the inherent projective geometry between the views. It only depends on the camera intrinsic parameters and the relative pose of the two cameras. Thus after the target is detected on the two cameras independently, epipolar geometry constraints between the cameras can be used to get data association results. In this way, the corresponding relationship of the candidate targets are confirmed and parts of false targets are removed.

Define $I_{1}=\left\{x_{1}^{1},x_{2}^{1},\ldots ,x_{m}^{1}\right\}$ and $I_{2}=\left\{x_{1}^{2},x_{2}^{2},\ldots ,x_{m}^{2}\right\}$ as the detection results of the first and second camera. The duty of the data association is to find the corresponding relationship between $x_{i}^{1}$ and $x_{j}^{2}$. Distance measurement is obtained by the symmetric transfer error between $x_{i}^{1}\left(i=1,2,\ldots ,m\right)$ and $x_{j}^{2}\left(i=1,2,\ldots ,n\right)$, can be defined as:

(5) $$d\left(x_{i}^{1},x_{j}^{2}\right)=d\left(x_{i}^{1},F^{T}x_{j}^{2}\right)+d\left(x_{j}^{2},Fx_{i}^{1}\right),$$

where F is the fundamental matrix between the two cameras. The matching matrix between two images is:

(6) $$D=\left[\begin{array}{cccc} d(x_{1}^{1},x_{1}^{2}) & d(x_{1}^{1},x_{2}^{2}) & \ldots & d(x_{1}^{1},x_{n}^{2})\\ d(x_{2}^{1},x_{1}^{2}) & d(x_{2}^{1},x_{2}^{2}) & \ldots & d(x_{2}^{1},x_{n}^{2})\\ \vdots & \vdots & \vdots & \vdots \\ d(x_{m}^{1},x_{1}^{2}) & d(x_{m}^{1},x_{2}^{2}) & \ldots & d(x_{m}^{1},x_{n}^{2}) \end{array}\right].$$

The global optimal matching result is obtained by solving the matching matrix *D* using Hungarian algorithm [36], which is taken as the final detection result.

***Target Localization:*** Supposing the world coordinate of the laser marker is *X*, the two camera parameter matrices are *P* and $P^{\prime }$, respectively. For the two detection images, supposing the image coordinates of the laser markers are *x* and $x^{\prime }$. Due to the measurement error, there are no points meet the equations x≅PX and $x^{\prime }≅P^{\prime }X$ strictly, and the image point is not satisfied to epipolar geometry constraint $x^{\prime T}Fx=0$.

A projective invariant binocular location method to minimize the re-projection error is presented here. The method is to find the exact solution to meet the minimum epipolar geometry constraint and re-projection error. Since the whole process only involves the projection of the space points and the distance of 2D image points, the method is projective invariant, which means that the solution is independent of the specific projective space.

In the corresponding images of the two cameras, the observation points are *x* and $x^{\prime }$ respectively. Supposing that the points near *x* and $x^{\prime }$ which precisely meet to epipolar geometry constraint are $\hat{x}$ and ${\hat{x}}^{\prime }$. Maximum likelihood estimation of the following objective function:

(7) $$C\left(\hat{x},{\hat{x}}^{\prime }\right)=d\left(x,\hat{x}\right)+d\left(x^{\prime },{\hat{x}}^{\prime }\right),$$

subject to ${\hat{x}}^{\prime T}F\hat{x}=0$, where d(∗,∗) is the Euclidean distance between image points.

We obtain initial value of *x* and $x^{\prime }$ by DLT algorithm firstly. Supposing $x≅P^{\prime }X$ and $x^{\prime }≅P^{\prime }X$, two equations $x^{\prime }PX=0$ and $x^{\prime }P^{\prime }X=0$ are obtained by the homogeneous relation. By expansioning the equations and we get:

(8) $$\left\{\begin{array}{c} x_{1}\left(p^{3T}X\right)-\left(p^{1T}X\right)=0\\ y_{1}\left(p^{3T}X\right)-\left(p^{2T}X\right)=0\\ x_{1}\left(p^{2T}X\right)-y_{1}\left(p^{1T}X\right)=0\\ x_{2}\left(p^{\prime 3T}X\right)-\left(p^{\prime 1T}X\right)=0\\ y_{2}\left(p^{\prime 3T}X\right)-\left(p^{\prime 2T}X\right)=0\\ x^{2}\left(p^{\prime 2T}X\right)-y^{2}\left(p^{\prime 1T}X\right)=0 \end{array}\right.,$$

where $p^{iT}$ is the i-th row of the matrix *P*, $p^{\prime jT}$ is the j-th column of matrix $P^{\prime }$. The homogeneous coordinate equations are $x={\left(x_{1},y_{1},1\right)}^{T}$ and $x^{\prime }={\left(x_{2},y_{2},1\right)}^{T}$. The formula for linear equations on *X* can be written as AX=0. Although each set of points correspond to the three equations, only two of them are linearly independent. Thus each set of points could provide two equations about *X*. The third equation is usually omitted when solving *X*. Thus A could be described as:

(9) $$A=\left[\begin{array}{c} x_{1}p^{3T}-p^{1T}\\ y_{1}p^{3T}-p^{2T}\\ x_{2}p^{\prime 3T}-p^{\prime 1T}\\ y_{2}p^{\prime 3T}-p^{\prime }2T \end{array}\right].$$

Since *X* is a homogeneous coordinate, only three degrees of freedom are scale-independent. The linear equation set AX=0 contains four equations, so the linear system actually is a over-determined system. To get the approximate solution of *X*, equation set AX=0 could be changed into the following optimization problem:

(10) $$\min_{x}∥AX∥,$$

subject to ∥X∥=1.

After the initial value $X_{0}$ of X is obtained from the above formula, LM algorithm is used for the iterative optimization to yield final localization results.

***Target Tracking:*** The Euclidean distance is used as the distance measurement in the 3D space. Define the historical target tracking result $T_{i}^{t}$(i=1,2,...,p) and current localization result $X_{j}^{t\;+\;1}$(j=1,2,...,q), the distance between them is computed by:

(11) $$d\left(T_{i}^{t},X_{j}^{t+1}\right)=\sqrt{{\left(x_{i}^{t}-x_{j}^{t\;+\;1}\right)}^{2}+{\left(y_{i}^{t}-y_{j}^{t\;+\;1}\right)}^{2}+{\left(z_{i}^{t}-z_{j}^{t\;+\;1}\right)}^{2}},$$

where $\left(x_{i}^{t},y_{i}^{t},z_{i}^{t}\right)$ and $\left(x_{j}^{t\;+\;1},y_{j}^{t\;+\;1},z_{j}^{t\;+\;1}\right)$ are space coordinates of $T_{i}^{t}$ and $X_{j}^{t\;+\;1}$. Thus the matching matrix between them is computed by:

(12) $$D_{t}^{t+1}=\left[\begin{array}{cccc} d(T_{1}^{t},X_{1}^{T\;+\;1}) & d(T_{1}^{t},X_{2}^{T\;+\;1}) & \ldots & d(T_{1}^{t},X_{q}^{T\;+\;1})\\ d(T_{2}^{t},X_{1}^{T\;+\;1}) & d(T_{2}^{t},X_{2}^{T\;+\;1}) & \ldots & d(T_{2}^{t},X_{q}^{T\;+\;1})\\ \vdots & \vdots & \vdots & \vdots \\ d(T_{p}^{t},X_{1}^{T\;+\;1}) & d(X_{p}^{t},X_{2}^{T\;+\;1}) & \ldots & d(x_{p}^{t},X_{q}^{T\;+\;1}) \end{array}\right].$$

The Hungarian algorithm is used to get the target tracking results from $D_{t}^{t\;+\;1}$.

## 3. Experiments and Discussion

### 3.1. Optical Imaging Experiments

We have compared several different kinds of light sources such as strong light flashlight, high intensity discharge lamp, halogen lamp, etc. Because of those light sources are sensitive to visible light and have a short irradiation range, we finally choose the near infrared laser lamp. In this section, we will present the comparison results of near infrared laser lamp and strong light flashlight.

**Figure 5:**
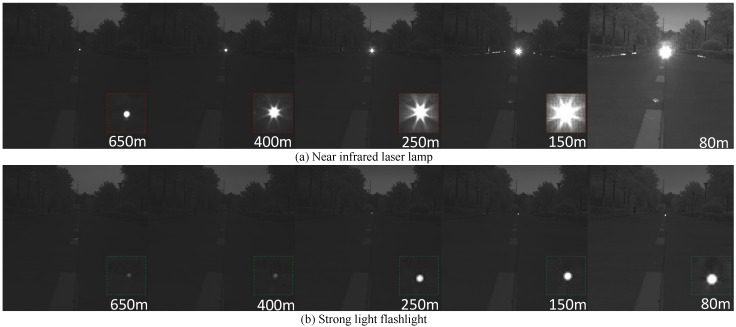
The comparison of the imaging at different distances.

We compare the quality of the imaging at different distances firstly. In this experiment, the near infrared laser lamp and the flashlight are placed in the same position at different distances. The experimental distance is from 80 m to 650 m. Figure 5 shows that the light spots of the strong light flashlight in the images are hard to find after 400 m, while the light spots of the near infrared laser lamp can still be seen clearly at 650 m.

**Figure 6:**
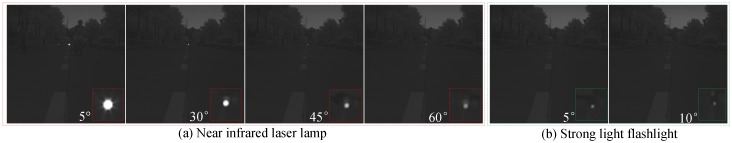
The comparison of the imaging from different angles at the distance of 150 m. (**a**) The near infrared laser lamp: from 0 degrees to 45 degrees; (**b**) The strong light flashlight: from 0 degrees to 10 degrees.

The system need to have a certain fault tolerance to the angle change. In this experiment, we place the light sources at the same position with the same directions firstly, and then adjust the horizontal rotation angle of the light source. We did this experiment at 150 m. As shown in Figure 6, the near infrared laser lamp can be detected robustly from 0 degrees to 45 degrees, while the strong light flashlight cannot be seen clearly when the angle is greater than 10 degrees.

From the above experiments, we can see that the near infrared laser lamp greatly meets the needs of the landing system. Cooperated with the infrared camera array and optical filter, a robust optical imaging system with long detection range is established.

### 3.2. Infrared Camera Array Calibration Experiments

The precision of the camera array parameters directly determines the localization accuracy. To verify, five reference points are selected near the center line of the runway to simulate the UAV position. Their space coordinates are measured by electronic total station as the ground truth. Then laser markers are placed to the positions of the reference points, of which the space coordinates are calculated based on the calibration results. In fact, this experiment could also be considered as a localization accuracy verification of UAV on the ground.

The experiment results are shown in Table 6. It is obviously to observe that the errors of *X* elements are much larger than *Y* and *Z* elements, while the errors of *Y* and *Z* remain below a limited threshold. The measurement errors gradually descend from far to near in *X* axis, and the precision has attained centimeter level within 200 m. Limited by the length of the runway, the maximum experimental distance is about 400 m. And in this experiment, the accuracy of the *Y* axis and the *Z* axis is controlled within the centimeter level. More importantly, we can see that a high localization accuracy has been attained in the last 200 m, which greatly meets the needs of the UAV automatic landing system.

**Figure 7:**
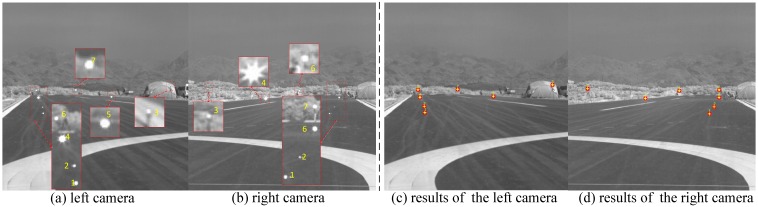
Verification of calibration results(red circles in (**c**) and (**d**) are real positions of the control points, the positions of yellow crosses are calculated by re-projection based on calibration parameters).

A practical experiment based on control points has also been conducted to verify the calibration results as shown in Figure 7. The calibration images taken by the two infrared cameras are shown in Figure 7a,b. As described previously, the positions of the control points are measured by electronic total station. The red circles in Figure 7c,d are real positions of the control points, which are marked by our detection algorithm. The world coordinates of control points are re-projected to the image coordinates based on calibration parameters, which are marked by yellow crosses as shown in Figure 7c,d. We can see that the red circles and yellow crosses are basic coincidence, which demonstrate the calibration accuracy of the intrinsic and external parameters effectively.

### 3.3. Target Detection and Localization Experiments

In order to improve the stability and robustness of the ground-based system, the landing system needs to be able to remove the false targets effectively. The removal of false targets is mainly reflected in three aspects. In the process of multi-camera collaborative detection based on the epipolar constrain, the false targets can be removed by the symmetric transfer error; In the process of the stereo vision localization of multi-camera, the false targets can be removed by the space motion track constraints of the UAV; In the process of the target tracking, the false targets can be removed by analyzing the motion directions and velocities of the candidate targets. In this way, the target can be detected correctly.

**Figure 8:**
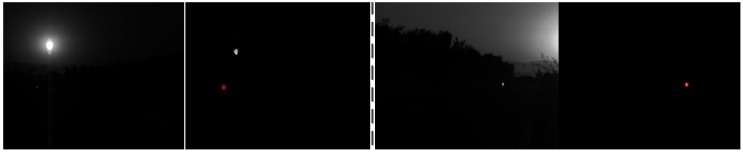
The detection results under sunlight.

**Figure 9:**
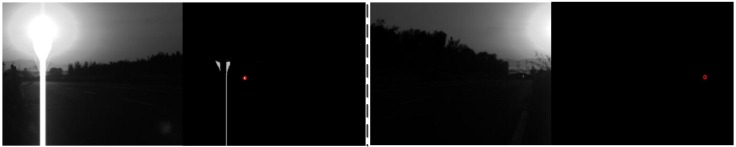
The detection results under sunlight with smear effect.

Figure 8 and Figure 9 shows the detection experiments under sunlight, even with smear effect in Figure 9, we can see that the targets are both detected correctly.

The detection accuracy of the near infrared laser lamp fixed on the UAV directly determines the accuracy of the target spatial localization. In this part, we will analyze the effects of the detection error on the localization accuracy. In this simulation experiment, we assume that the camera parameters are already known. During the landing phase, the point is projected to the image through the camera matrix, and Gaussian random noise with a mean of zero is added on the projected point. And then we get the localization result and analyze the localization accuracy. Table 7 gives the average error results of 1000 times simulation experiments with different standard deviation of Gaussian noise.

In Table 7, the standard deviation of the Gaussian noise is set to 0.1 pixels, 0.2 pixels and 0.5 pixels in turn. The error of the three axes decreases with the decrease of the distance. The error in *X* axis is the largest, and the error in *Y* axis and *Z* axis remains a high accuracy. When the standard deviation of the Gaussian noise is set to 0.5 pixels, the error in three axes is the largest. However, in the last 100 m, the error in *X* axis is less than 0.5 m and the error in *Y* axis and *Z* axis is within the centimeter level. We can see that when the target detection accuracy is less than 0.5 pixels, the localization accuracy could meet the requirements of the landing system.

We have performed extensive automatic landing experiments in several places successfully. In order to enlarge the field of view, we usually choose the runway whose width is more than 15 m. The field of view is usually determined by the width and length of the runway. As described previously, the two infrared cameras are located on the two sides of the runway respectively to capture flying aircraft images. One of basic principles is that the public field of view should cover the landing area of the UAV. In the actual experiment, the landing area is usually already known, thus it is easy to make the infrared camera array cover the landing area. And to ensure the accuracy, the imaging of the landing area should be close to the image center, especially for the last 200 m. The detection range changes with the baseline. With the baseline of 20 m, the minimum detection range is about 25 m and the maximum detection range is over 1000 m. The UAV takes off and cruise at an altitude of 200 m using the DGPS system. Once the UAV is detected and the error is acceptable, the UAV is guided to land by our ground-based vision system.

### 3.4. Real-Time Automatic Landing Experiments

It is important to ensure the safety and reliability of the UAV automatic landing system, therefore, the verification of the UAV real-time localization accuracy is necessary. Thus, we compared the localization accuracy with DGPS measurements. The DGPS data is produced by SPAN-CPT module as shown in Table 8. SPAN-CPT is a compact, single enclosure GNSS + INS receiver with variety of positioning modes to ensure the accuracy. The IMU components within the SPAN-CPT enclosure are comprised of Fiber Optic Gyros (FOG) and Micro Electromechanical System (MEMS) accelerometers, etc. The tight coupling of the GNSS and IMU measurements delivers the most satellite observations and the most accurate, continuous solution possible. In our experiments, we choose the RT-2 module and its horizontal position accuracy is 1 cm + 1 ppm. During the process of landing, the localization results of the ground-based system are uploaded to the UAV control center through wireless data chain, and then the received data and current DGPS measurement results will be saved at the same time by the UAV control center, of which the maximum data update rate is 200 HZ. By analyzing the stored localization data after UAV landing, the localization accuracy can be verified.

Airborne DGPS measurement data is usually defined in the geographic coordinate system, while the vision measurement data is defined in the ground world coordinate system. To analyze the errors between them, the conversion between the DGPS coordinates and the world coordinates is necessary. To ensure the accuracy of coordinate conversion, three points are selected, one is the origin of the world coordinates, and the others are far long the runway (e.g., 200 m). Their coordinate information such as longitude, latitude and altitude is measured by the DGPS measurement module. The world coordinates of the three points are measured by the electronic total station. The direction of the runway can be obtained by the two points that far along the runway, computed with the origin coordinate, the conversion relationship between the coordinate systems can be finally determined.

**Figure 10:**
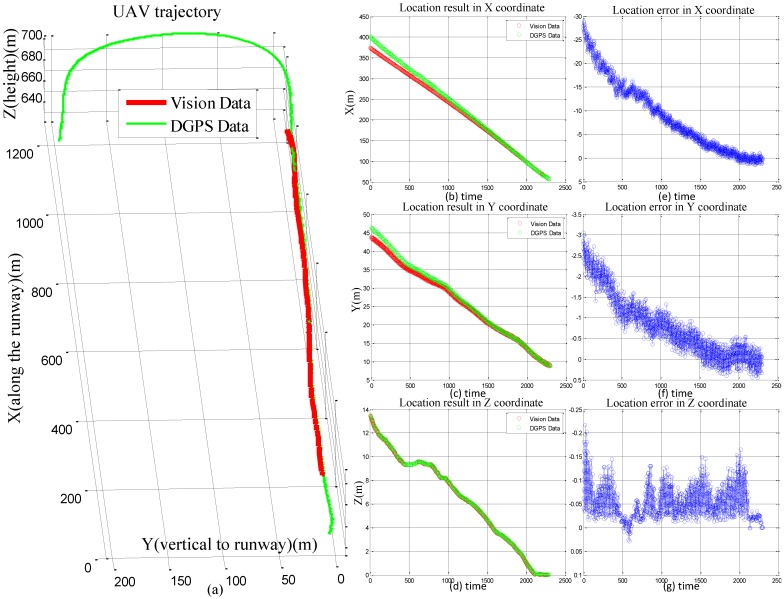
Comparison of DGPS and vision data. (**a**) The UAV trajectories of vision-based method and DGPS method; (**b**–**d**) The location results in *X*, *Y* and *Z* coordinates. (**e**–**g**) The location errors in *X*, *Y* and *Z* coordinates.

We compared the localization results with DGPS in Figure 10a. We can see that the detection range is over 1000 m and the data generated by our system are coincident with the DGPS data. The accuracy in *Z* axis is the most important factor to real UAV automatic landing. On the contrary, the accuracy in *X* axis has the minimal influence on its automatic landing because of the long runway. And the limited width of the runway also needs a high localization accuracy at *Y* axis. In this experiment, we refer the data from DGPS as the ground truth, the absolute errors in *X*, *Y*, *Z* are evaluated as shown in Figure 10e–g. The errors of *X* elements are the largest compared with *Y* and *Z* elements. However, the errors of *X* and *Y* elements are gradually decreased during the landing phase. At the last 200 m, The location errors in *X* and *Y* coordinates have reduced to below 5 and 0.5 m, respectively. Both of which have achieved a high accuracy. And at the last 100 m, the localization results in *X* and *Y* coordinates nearly are the same with DGPS. To avoid the crash, a high precision estimation of altitude should be guaranteed. We have achieved an impressive localization result in *Z* axis, in which the error is less than 0.22 m during the whole landing process. The measurement precisions in the whole landing process completely meet the requirements of the UAV control center.

**Figure 11:**
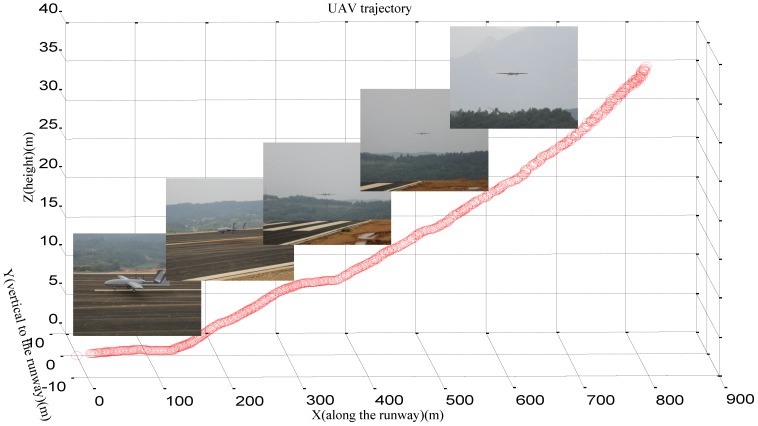
Landing trajectory away from 800 m.

Figure 11 shows one of the landing trajectories generated by our system and several landing poses of the UAV are presented here. Under the control of our system, the poses of the UAV could remain steady during the whole process of the decline. When the UAV was controlled by our ground-based system, the GPS jammer was turned on. Thus, in this experiment, the UAV was controlled from 820 m in the GPS-denied environment and successful landing, we can see that the trajectory is smooth and complete.

## 4. Conclusions

This paper described a novel infrared camera array guidance system for UAV automatic landing in GPS-denied environment. We overcome the shortcomings of the traditional GPS method which is easily blocked, etc. After an optical imaging system is designed, a high precision calibration method for large scene based on electronic total station is provided. The feasibility, accuracy has been verified through real-time flight experiments without GPS, and the results have identified that the control distance of our system is over 1000 m and a high landing accuracy has been achieved.

## Figures and Tables

**Table 1:** The near infrared laser lamp parameters.

Near Infrared Laser Lamp	Specification	Parameter
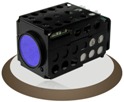	wavelength	808 ± 5 nm
	illumination distance	more than 1000 m
	weight	470 g
	working voltage	DC12V ± 10%
	maximum power consumption	25 W
	operating temperature	$0{\;}^{{}^{\circ}}$C∼50 ${}^{{}^{\circ}}$C

**Table 2:** The camera parameters.

Camera	Specification	Parameter
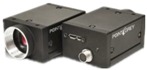	sensor	CMOSIS CMV4000-3E12
	maximum resolution	2048×2048
	maximum frame rate	2048×2048 at 90 fps
	interface	USB 3.0
	maximum power consumption	4.5 W
	operating temperature	$-20{\;}^{{}^{\circ}}$C∼50 ${}^{{}^{\circ}}$C

**Table 3:** The camera lens parameters.

Camera Lens	Specification	Parameter
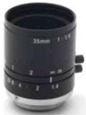	focal length	35 mm
	F No.	1.4
	range of WD	110 mm–∞
	maximum magnification	0.3×
	TV distortion	-0.027%
	filter pitch	M46P = 0.75
	maximum compatible sensor	1.1

**Table 4:** The Near-IR interference bandpass filter parameters.

Filter	Specification	Parameter
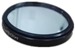	useful range	798∼820 nm
	FWHM	35 nm
	tolerance	±5 nm
	peak transmission	≥85%

**Table 5:** The electronic total station parameters.

Electronic Total Station	Specification	Parameter
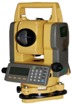	field of view	$1^{{}^{\circ}}30^{{}^{\prime }}$
	measuring distance	2000 m
	ranging accuracy	±(2 mm + 2 ppm)
	angle measurement accuracy	$\pm 2^{{}^{\prime \prime }}$
	ranging time	1.2 s
	operating temperature	$-20{\;}^{{}^{\circ}}$C∼50 ${}^{{}^{\circ}}$C

**Table 6:** The calibration accuracy analysis.

Serial	Reference Points (m)			Localization Results (m)			Errors (m)		
Number	*X*(m)	Y(m)	Z(m)	$X^{\prime }$	$Y^{\prime }$	$Z^{\prime }$	ΔX	ΔY	ΔZ
1	64.363	0.012	2.072	64.274811	0.012149	2.060729	−0.088188	0.000149	−0.011271
2	102.898	−0.068	2.185	102.961128	−0.066252	2.164158	0.063126	0.001748	−0.020842
3	198.018	−0.141	2.615	197.970352	−0.113468	2.613832	−0.047653	0.027532	−0.001168
4	303.228	−0.324	3.049	300.387817	−0.283089	2.991707	−2.840179	0.040911	−0.057293
5	395.121	−0.567	3.427	396.371521	−0.573597	3.456094	1.250519	−0.006597	0.029094

**Table 7:** The effect of the detection error on localization accuracy.

Pixel Errors	0.5 Pixels			0.2 Pixels			0.1 Pixels		
**Distance (m)**	X(m)	Y(m)	Z(m)	X(m)	Y(m)	Z(m)	X(m)	Y(m)	Z(m)
10	0.0905	0.0092	0.0091	0.0367	0.0037	0.0037	0.0180	0.0018	0.0018
20	0.1173	0.0105	0.0104	0.0479	0.0042	0.0042	0.0225	0.0021	0.0021
30	0.1430	0.0113	0.0123	0.0550	0.0047	0.0049	0.0285	0.0024	0.0024
40	0.1783	0.0128	0.0137	0.0714	0.0051	0.0056	0.0339	0.0026	0.0027
50	0.2078	0.0139	0.0163	0.0810	0.0056	0.0060	0.0393	0.0027	0.0031
100	0.4099	0.0198	0.0288	0.1709	0.0080	0.0120	0.0839	0.0039	0.0062
150	0.7081	0.0248	0.0503	0.2846	0.0102	0.0205	0.1418	0.0053	0.0101
200	1.1051	0.0305	0.0790	0.4307	0.0129	0.0302	0.2099	0.0065	0.0155
300	2.0075	0.0422	0.1501	0.7960	0.0167	0.0591	0.3989	0.0087	0.0294
400	3.1424	0.0549	0.2423	1.3165	0.0217	0.1013	0.6523	0.0105	0.0497

**Table 8:** The SPAN-CPT parameters.

The SPAN-CPT	Specification	Parameter
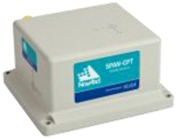	horizontal positon accuracy (RT-2 module)	1 cm + 1 ppm
	horizontal positon accuracy (single point)	1.2 m
	heading accuracy	$0.03^{{}^{\circ}}$
	bias (gyroscope)	±20 ${}^{{}^{\circ}}/h$
	bias stability (gyroscope)	±1 ${}^{{}^{\circ}}/h$
	bias (accelerometer)	±50 mg
	bias stability (gyroscope)	±0.75 mg
	speed accuracy	0.02 m/s
	weight	2.28 kg

## Supplementary Materials

The following are available online at http://www.mdpi.com/1424-8220/16/9/1393/s1.
